# Bacteriology of Aural Furuncles

**Published:** 1887-10

**Authors:** B. Lowenberg

**Affiliations:** Paris, France


### *THE TREATMENT AND THE BACTERIOLOGY OF AURAL FURUNCLES.*

By Dr. B. LOWENBERG, of Paris, France.

In 1880 and 1881, I published the results of my researches on the practical nature of aural furuncles, together with the theoretical and practical applications, the substance of which can be given as follows:

1. Boils are caused by an affection from outward, viz., through the ducts of the cutaneous follicles.

2. The successive outbreak of furuncles on the same individual takes place by auto-contagion, that is, through transport of the cocci upon the skin.

3. Infection from one person to another is possible, and originates from the same process as in No. 2.

These fundamental points have led me to a plan of treatment for boils and for furunculosis in general. I shall now expose this method with regard to the furuncles of the external ear, together with the results hitherto obtained.

My course of treatment is about the same as the one formerly proposed by me for otorrhœa, that is, the use of an over-saturated solution of one part of extremely fine powder of boracic acid to five parts of strong, even absolute, alcohol. This compound I use in instillations into the meatus, to be repeated three to four times a day.

As long as a boil is not yet opened, a simple saturated alcoholic solution of boracic acid is sufficient, but when pus is already discharging, I prefer the over-saturated solution, as it deposes a certain amount of boracic powder, dissolving gradually in the pus and thus exercising a continual anti-bacterial action.

Alcohol, besides its efficaciousness against micro-organisms, is moreover destinated to facilitate the penetration of the compound into the ducts of the follicles, the seat of the disease. The fatty lining and contents of these capillary canals oppose, according to physico-chemical laws, a resistance to the entering of watery liquids, while alcohol, according to its affinity to fats, easily penetrates.

Incision of boils certainly sometimes facilitates this course of treatment, but it is often very difficult to practice it just so as to divide the follicle-ducts, which seems to me the desideratum.

Cocaine, though applied upon the epidermis, often procures passing relief.

RESULTS.

An early application of the saturated solution of boracic acid in strong alcohol often arrests the boils; even in the cases where this abortive treatment should fail, the perseverant use of the over-saturated solution always stops the otherwise nearly unavoidable succession of boils, originated by auto-contagion, as I have called it (*loc. cit.*). These results seem to me of great importance, firstly, because aural furuncles are known to be extremely painful; secondly, because, according to my experience, the longer this local furunculosis lasts the greater is the tendency of the boils to form in parts situated nearer and nearer the drum, and, consequently, to prove more and more painful. These results, to the best of my knowledge, have not been obtained before my researches.

Many female patients suffer, often for years, from aural boils arising before or during each menstrual period, a fact, an explanative theory of which will be found in my paper. In such cases, my treatment arrests these boils, or, at least, prevents their return. Nay, their formation may even be successfully prevented by a prophylactic use of this treatment begun before the catamenial epoch.

The same results can be obtained with persons who are regularly attacked by this affection in spring or fall.

BACTERIA IN EAR-FURUNCLES.

I have undertaken bacteriological researches in a certain number of cases of still unopened boils of the meatus. In each case, I first syringed this canal and then filled it for ten minutes with a lukewarm solution of bichloride of mercury ($\frac{1}{2000}$). A small parcel of the pus was inoculated into agar-agar, or nutriment gelatine, and plate cultivations were made use of the whole.

I obtained the following results: The micro-organism most frequently found was staphylococcus albus, which was absent in only one case, then came staphylococcus aureus and sometimes staphylococcus citreus. Only in one case all these three staphylococci were traced together.

These results differ from those obtained by my friend N. Kirchner, from Würzburg, who only found staphylococcus albus.

